# Shear bond strength of Biodentine, ProRoot MTA, glass ionomer cement and composite resin on human dentine *ex vivo*

**DOI:** 10.1186/s13005-015-0071-z

**Published:** 2015-04-19

**Authors:** Markus Kaup, Christoph Heinrich Dammann, Edgar Schäfer, Till Dammaschke

**Affiliations:** Department of Operative Dentistry, Westphalian Wilhelms-University, Albert-Schweitzer-Campus 1, building W 30, 48149 Münster, Germany; Central Interdisciplinary Ambulance in the School of Dentistry, Albert-Schweitzer-Campus 1, building W 30, 48149 Münster, Germany

**Keywords:** Biodentine, Composite resin, Glass ionomer cement, ProRoot MTA, Shear bond strength

## Abstract

**Introduction:**

The aim of this study was to compare the shear bond strength of Biodentine, ProRoot MTA (MTA), glass ionomer cement (GIC) and composite resin (CR) on dentine.

**Methods:**

120 extracted human third molars were embedded in cold-cured-resin and grinned down to the dentine. For each material 30 specimens were produced in standardised height and width and the materials were applied according to manufacturers´ instructions on the dentine samples. Only in the CR group a self-etching dentine-adhesive was used. In all other groups the dentine was not pre-treated. All specimens were stored at 37.5 °C and 100% humidity for 2d, 7d and 14d. With a testing device the shear bond strength was determined (separation of the specimens from the dentine surface). The statistical evaluation was performed using ANOVA and Tukey-test (p < 0.05).

**Results:**

At all observation periods the CR showed the significant highest shear bond strength (p < 0.05). After 2d significant differences in the shear bond strength were detectable between all tested materials, whereby CR had the highest and MTA the lowest values (p < 0.05). After 7d and 14d the shear bond strengths of MTA and Biodentine increased significantly compared to the 2d investigation period (p < 0.05). Biodentine showed a significantly higher shear bond strength than MTA (p < 0.05), while the difference between Biodentine and GIC was not significant (p > 0.05).

**Conclusions:**

After 7d Biodentine showed comparable shear bond values than GIC, whereas the shear bond values for MTA were significantly lower even after 14d. The adhesion of Biodentine to dentine surface seams to be superior compared to that of MTA.

## Introduction

In the early 1990s the development of mineral trioxide aggregate (MTA) introduced a new class of dental calcium silicate cements (CSCs) based on Portland cement, originally developed as a perforation repair material. Since then it has been widely used for repair-purposes of furcations and root canals, as a root-end filling material, for direct and indirect pulp-capping and the treatment of extern and intern resorptions with high success rates [1-3].

Beside a long setting time the major drawbacks of MTA are its relatively low compression and flexural strength, which are lower than those of dentine [4]. These factors are limiting the field of application to low stress-bearing areas [5]. Hence, MTA can not be used e.g. as base, base build-up, core material or as temporary restoration.

This triggered the development of new formulas of calcium silicate-based cements to overcome these drawbacks and keep the advantages. Biodentine (Septodont, St.-Maur-des-Fossés, France) can be considered as an outcome of this process. This calcium silicate based cement uses synthetically created pure raw materials in comparison to the impure base materials of MTA. Biodentine mainly consists of tri- and dicalcium silicate as the main material, calcium carbonate as filler for enhancing mechanical properties and accelerating the hardening of the cement and zirconium dioxide as a radiopacifier [5-7]. In contrast to MTA, which uses only distilled water for setting, Biodentine uses a mix of distilled water, calcium chloride and a hydrosoluble polymer. Calcium chloride acts as an accelerator of the setting reaction [8]. The hydrosoluble polymer reduces the necessary water of the reaction [7]. With these improvements of the composition the initial setting time of Biodentine (12 min) is much lower than that of MTA (180 min) [6]. Biodentine is bioactive and biocompatible like MTA [6,9] and possesses the same good sealing abilities than other CSCs [7].

The microhardness, flexural and compressive strength of Biodentine are higher than those of other CSCs and more comparable to dentine [7,10]. Thus, Biodentine can also be used as an alternative to glass ionomer cements (GICs) in restorative dentistry [11].

Besides having a fast setting time and a high compressive strength any material used for posterior fillings must also have the ability to create a bond between the material and the dentine like GICs or dentine adhesives. A material used as a base or base build-up should provide an adequate seal, be able to prevent leakage and remain in place under dislodging forces, such as chewing pressure or the application of other restorative material, thus having adhesive properties to dentine. Hence, the bond strength of restorative materials is an important factor in clinical practice.

However, to the best of our knowledge there is no study available, which compares the shear bond strength of Biodentine, MTA, glass ionomer cement, and composite resin on a planar dentine surface in the same experimental setup. Thus, the aim of this *ex vivo* study was to determine the shear bond strength of Biodentine on dentine after 2, 7, and 14 days and to compare this values with another CSC (ProRoot MTA; Dentsply-Maillefer, Ballaigues, Switzerland), a glass ionomer cement (ChemFil rock; Dentsply, Konstanz, Germany) and a composite resin for bulk filling (X-tra base universal; VOCO, Cuxhaven, Germany). All these different materials are recommended to replace missing dentine in restorations or endodontic therapies.

The null-hypotheses of this study were that (i) the shear bond strengths to dentine of Biodentine and MTA are equal and that (ii) Biodentine and MTA possess lower shear bond strengths GIC and composite resin in combination with a dentine adhesive.

## Methods

120 human third molar teeth were stored in sterile saline solution directly after extraction and refrigerated at 5 °C for less than two weeks. The handling of all human samples followed strictly the “Declaration of Helsinki“. To produce plane-parallel samples the molar teeth were embedded in a cold-curing resin (Technovit 4071; Kulzer, Wehrheim, Germany) and ground with sandpaper (1200 grain) down to the dentine surface.

An impression of a columnar metal blank with a diameter of 3.28 mm and height of 4 mm was taken with polyether rubber impression material (Dimension Penta H and Impregum Garant L DuoSoft; 3 M ESPE, Seefeld, Germany). This negative form was used to ensure standardisation of samples. The mould made of polyether rubber was placed on the dentine samples and was completely filled with the particular test material avoiding any air entrapments, voids or gaps. The cross sectional area of the specimens was 8.5 mm^2^ and the test material had complete contact to the dentine surface without touching the enamel. All plane-parallel dentine samples were rinsed with distilled water and air dried directly before the application of the test materials.

ProRoot MTA, Biodentine, ChemFil rock and X-tra base universal were strictly handled in accordance with the manufactures´ instructions. Thus, for ProRoot MTA, Biodentine, and the GIC ChemFil rock the dentine was not further treated before application. After placing the polyether rubber mould on the dentine surface it was completely filled with the appropriate material with the aid of a cement plugger.

Only in the composite resin group the dentine was pre-treated with the recommended self-etching dentine adhesive (Futurabond DC Single Dose; VOCO, Cuxhaven, Germany). After light-curing (Elipar Highlight; 3 M ESPE, Seefeld, Germany) the X-tra base universal composite resin was put in the mould and light-cured by increment technique.

30 specimens were produced from every test material for shear bond testing after two, seven and 14 days (n = 10 per day) and stored in an incubator (U 29; Memmert, Schwabach, Germany) at 37.5 °C and 100% humidity. The polyether rubber moulds were removed from all specimens so that only the cylindrical test material adhered perpendicular to the dentine surface.

The shear bond strength was evaluated with the universal testing device LF Plus (Lloyd Instruments, Bognor Regis, Great Britain). All specimens were mounted in a metal mould which served as drive surface for a metal plunger. This plunger overlaid the specimen surface and touched the cylindrical test material at the contact with the dentine surface in a right angle (90°). The testing device moved with a defined feed speed of 1 mm/min towards the plunger. The shear bond strength needed to separate the test materials from the dentine surface was calculated with a special software program (Nexygen Version 4.5; Lloyd Instruments, Bognor Regis, Great Britain). The values were statistically analysed using analysis of variance (ANOVA) and post-hoc Tukey-test (p < 0.05).

The bonding failure modes of the restorative materials to the dentine surface were evaluated by using a laser scanning microscope (BZ 9000, Keyence, Osaka, Japan):

1. adhesive failure that occurred at the filling material and dentine interface
2. cohesive failure within the filling material
3. mixed failure mode

## Results

### Shear bond strength

The values of the shear bond strength and the statistical evaluation are given in Table 1. At all observation periods ProRoot MTA showed the lowest shear bond strength of all tested materials. After two days a slight touch with the metal plunger was effectual to detach all ProRoot MTA samples from the dentine surface. Thus, shear bond strength was not measurable and set to be “0”. Although the dentine bonding of ProRoot MTA increased over time after 14 d still 20% of the specimens showed no measurable bonding to the dentine surface. The shear bond strength for Biodentine after 2 d was nearly 3 MPa and tripled within one week to more than 9 MPa. The GIC ChemFil rock reached a shear bond strength of about 10 MPa after two days which was comparable to the values determined after one and two weeks. Hence, an in- or decrease of the values did not occur over time. The composite resin X-trabase universal in combination with the dentine adhesive Futurabond DC showed mean shear bond strengths of about 30 MPa with no significant changes over time.

**Table 1:** **Shear bond strength (mean and standard deviation) of the tested materials on dentine after 2 d, 7 d and 14 d**

**Material**	**2 d**	**7 d**	**14 d**
ProRoot MTA	0.0^**a**^	0.85 (±1.42)^a*^	4.96 (±4.54)^a**^
Biodentine	3.14 (±1.09)^**b**^	9.75 (±2,19)^b*^	9.34 (±1.01)^b*^
ChemFil rock (GIC)	10.92 (±5.69)^**c**^	9.73 (±4.03)^**b**^	9.94 (±1.44)^**b**^
X-trabase (composite resin) **+**	32.74 (±4.66)^**d**^	32.75 (±4.61)^**c**^	30.48 (±4.43)^**c**^
Futurabond DC (dentine adhesive)			

Values with the same superscript letters were not statistically different at p = 0.05.

* = statistically significant difference of the shear bond strength values in comparison to the values at the 2 d observation period (p < 0.05).

** = statistically significant difference of the shear bond strength values in comparison to the values at the 7 d observation period (p < 0.05).

All values are given in MPa. The statistical evaluation was performed using ANOVA and Tukey-test (p < 0.05).

The statistical evaluation revealed that the composite resin in combination with a dentine adhesive had the significantly highest shear bond strength of all tested materials at all observation periods (p < 0.05). After two days significant differences between all materials were obtained, while the composite resin in combination with a dentine adhesive had the highest and ProRoot MTA the lowest values (p < 0.05). After one week Biodentine showed a significantly higher shear bond strength than ProRoot MTA (p < 0.05), while the difference between Biodentine and GIC was not significant (p > 0.05). The shear bond strength of ProRoot MTA and Biodentine increased significantly compared to the 2 d observation period (p < 0.05). Comparable results were obtained after two weeks. The shear bond values for ProRoot MTA further increased as the values after 14 d they were significantly higher than those after 2 d and 7 d (p < 0.05), whiles no significant changes were detected in the Biodentine group between the 7 d and 14 d observation period (p > 0.05).

### Failure mode

**Figure 1:**
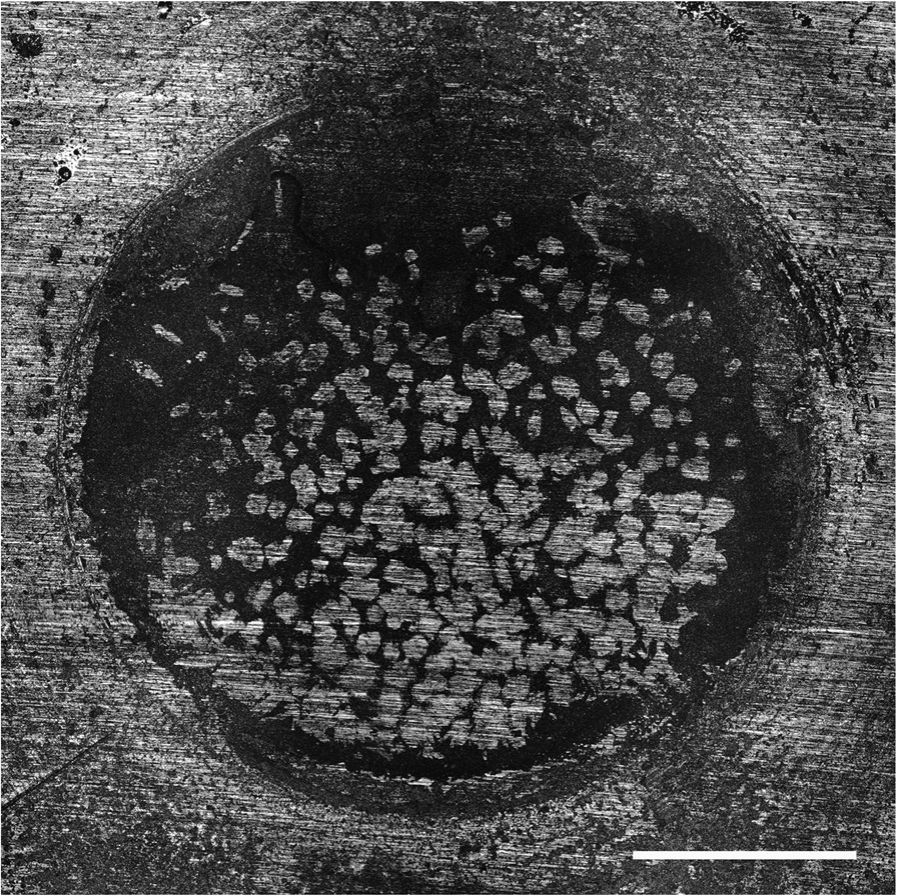
Example of a mixed failure mode of ProRoot MTA on dentine after 14 d. Original magnification x5. Bar represents 1 mm.

**Figure 2:**
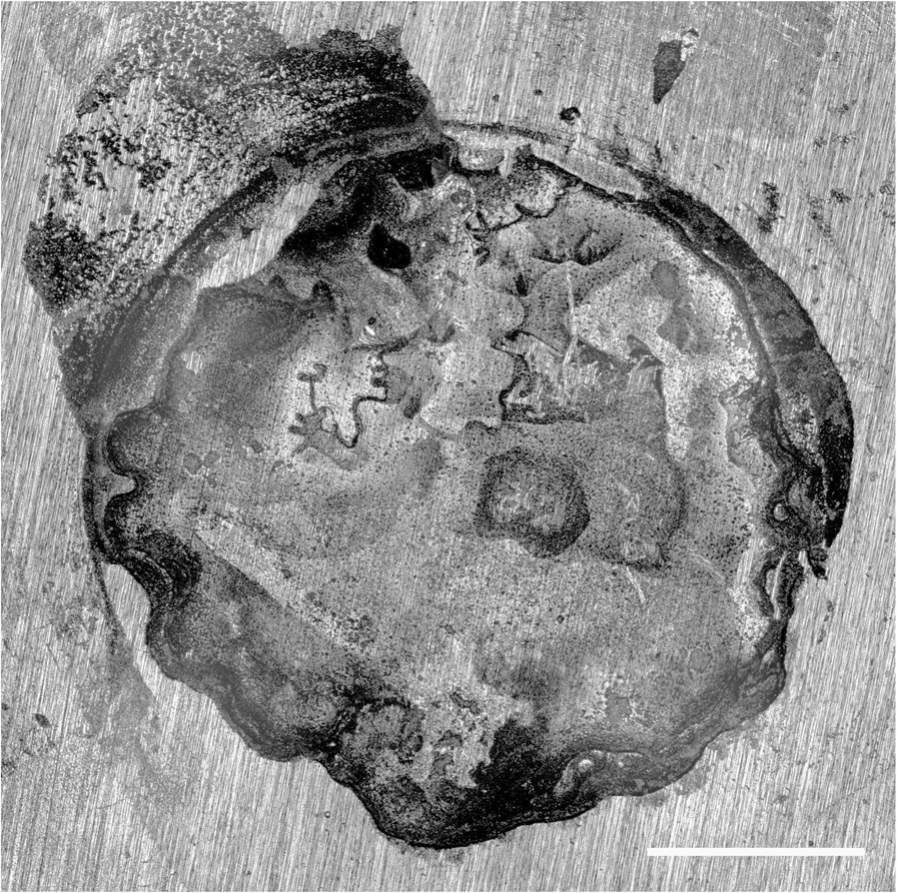
Example of a cohesive failure mode of Biodentine on dentine after 14 d. Original magnification x5. Bar represents 1 mm.

**Figure 3:**
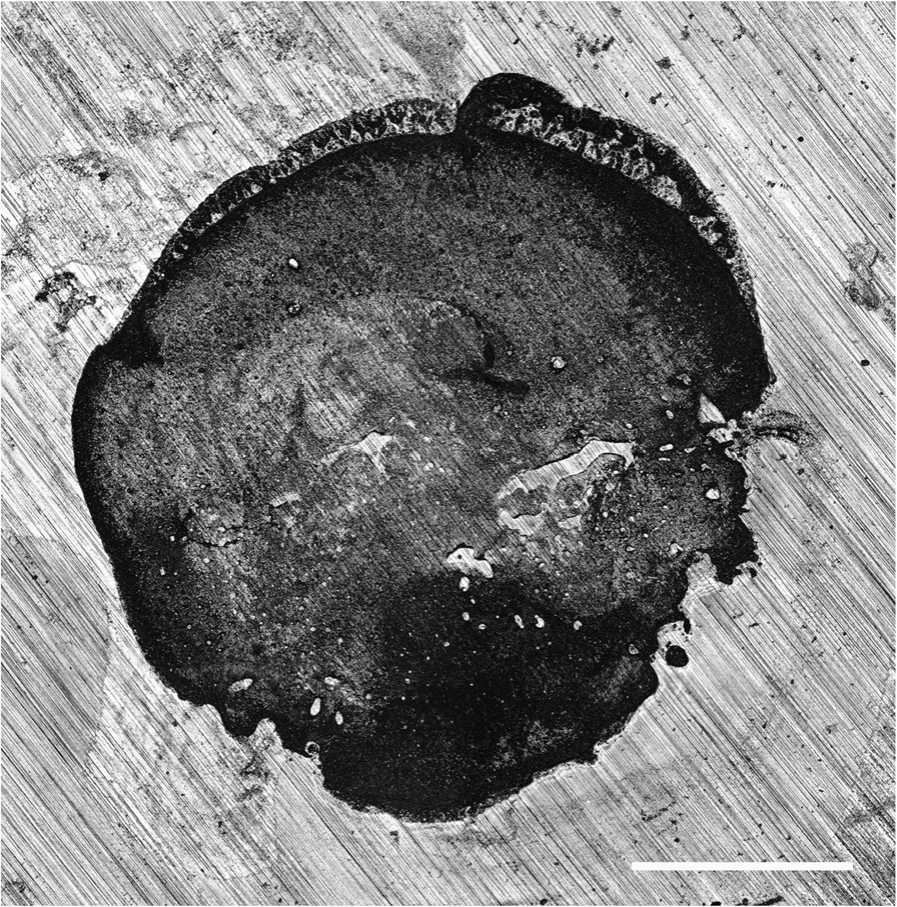
Example of a cohesive failure mode of ChemFil rock on dentine after 14 d. Original magnification x5. Bar represents 1 mm.

**Figure 4:**
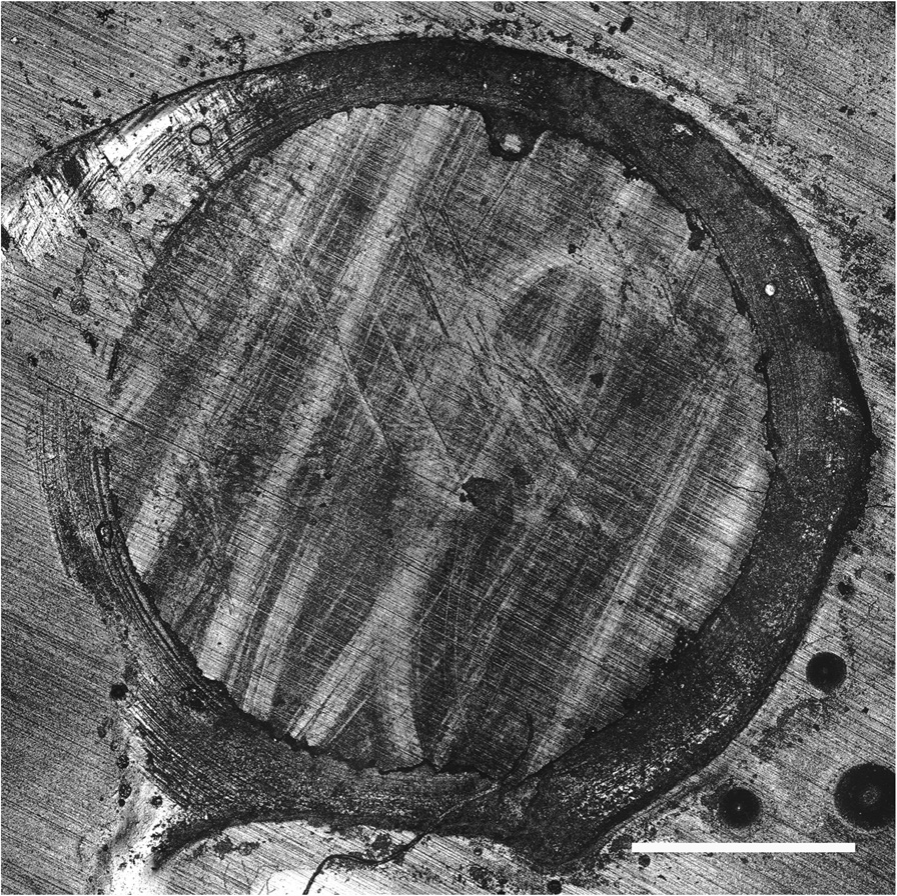
Example of an adhesive failure mode of X-trabase (composite resin) in combination with Futurabond DC (dentine adhesive) on dentine after 14 d. Original magnification x5. Bar represents 1 mm.

In the ProRoot MTA group 63.33% of the specimens showed an adhesive, 13.33% a cohesive and 23.33% a mixed failure mode (Figure 1), whereas in the Biodentine group a cohesive failure mode was noticed in 56.66% of the specimens and an adhesive failure mode occurred in 43.33% of the cases (Figure 2). In GIC samples the failure modes was mainly cohesive (93.33%) and in a minor part a mixture between an adhesive and cohesive failure (6.66%) (Figure 3). The composite resin and dentine adhesive showed an adhesive failure mode in 66.66% and a mixture between an adhesive and cohesive failure mode in 33.33% of the samples (Figure 4). Detailed results are given in Table 2.

**Table 2:** **Failure mode of ProRoot MTA (MTA), Biodentine (BD), ChemFil rock (GIC) and X-trabase (composite resin) in combination with Futurabond DC (CR) on dentine after 2 d, 7 d and 14 d**

**sample no.**	**MTA 2 d**	**MTA 7 d**	**MTA 14 d**	**BD 2 d**	**BD 7 d**	**BD 14**	**GIC 2 d**	**GIC 7 d**	**GIC 14 d**	**CR 2 d**	**CR 7 d**	**CR 14 d**
1	1	1	1	1	1	1	2	2	2	1	1	1
2	1	1	1	1	1	1	2	2	2	1	1	1
3	1	1	2	1	1	1	2	2	2	1	1	1
4	1	1	2	1	2	2	2	2	2	1	1	1
5	1	1	2	1	2	2	2	2	2	1	1	1
6	1	1	2	1	2	2	2	2	2	3	1	3
7	1	1	3	1	2	2	2	2	2	3	1	3
8	1	3	3	2	2	2	2	2	2	3	1	3
9	1	3	3	2	2	2	2	3	2	3	1	3
10	1	3	3	2	2	2	2	3	2	3	1	3

1 = adhesive failure mode that occurred at the filling material and dentine interface.

2 = cohesive failure mode within the filling material.

3 = mixed failure mode.

## Discussion

### Shear bond strength measurements

Theoretically, bond strength is defined as the interfacial adhesion between substrate and the bonded material, mediated by an adhesive layer. In practice, this is often not the case, and instead, fractures may take place in the bond material, the substrate, or both and may extend beyond the initial bonded area. What is actually being measured is the fracture force of a bonded system for a particular method of load application and should only cautiously be interpreted as bond strength [12]. Hence, the failure modes of e.g. GIC [12,13] and Biodentine [14,15] to dentine were largely cohesive within the cement rather than at the interface. Therefore, the rigidity of the material has a significant influence on the interpretation of bond strength [12] and thus the true clinical bond to dentine is probably different from the data given here.

Another important point to be noted is that the materials were not tested immediately after their initial setting, which is the real clinical scenario where the restored teeth are immediately subjected to masticatory stress. Here the samples were stored without any load up to 14 d before loading, which may have influenced the results. It has also to be kept in mind that the present study was performed using sound and healthy dentine. Thus, in cariously affected dentine the shear bond strength may be lower.

Hence, laboratory-generated shear bond values should be interpreted and transferred to the clinical situation with some caution [12].

### Bonding of CSCs to dentine

If the tricalcium silicate powder of a CSC is mixed with water a calcium-silicate-hydrate as well as calcium hydroxide is formed, which provides a high alkaline pH [6]. This is in contrast to most other dental cements like GIC, which are highly acidic during the setting reaction [16]. The exact mechanism regarding the bonding of CSCs to dentine is still unclear. Discussed is a chemical bond as well as a micromechanical anchorage via cement tags in the dentinal tubules [16-19]. E.g. after placement of MTA on dentine, hydroxyapatite crystals nucleate and grow, filling the microscopic space between MTA and the dentine surface. Initially this seal is mechanical. Over time, the reaction between hydroxyapatite and dentine leads to a chemical bonding [19]. Hence, MTA appeared to bond chemically to dentine via diffusion-controlled reaction between its apatitic surface and dentine, forming an adherent interfacial layer that was firmly attached to the dentin wall [19]. It was shown that MTA trigger the precipitation of carbonated apatite, promoting a controlled mineral nucleation on dentine that was observed as the formation of an interfacial layer with tag-like structures [18]. This mechanism, theoretically, could initially lead also to the retention of the cement by the dentine through a micromechanical bonding system [20]. The alkaline Biodentine may induce a caustic denaturation and permeability of the organic collagen component of interfacial dentine [16]. Hence, for Biodentine a recent study showed the formation of intra-tubular tags in conjunction with an interfacial mineral interaction layer referred to as the “mineral infiltration zone” [16]. The interfacial layer formed between Biodentine and dentine may be comparable to that formed between dentine and MTA [21]. In contrast to Atmeh et al. [16] the migration of ions into dentine was not shown for Biodentine by Gjorgievska et al. [21] This leads to the conclusion that the adhesion of Biodentine is mainly micromechanical, and not ion-exchange based. Nevertheless, Biodentine showed excellent adaptability toward dentine [21].

### Comparison to other Biodentine studies

To the best to our knowledge this is the first study that determined the shear bond strength values of Biodentine to a planar prepared coronal dentine surface. Nevertheless, the results of the present study are in the same range (6.2 MPa to 9.1 MPa) than push-out bond strength values [14,15,22-24]. However, the comparison of push-out bond strength of different CSCs with the results of the present study should be interpreted with some caution due to the different subject and methodology used in previous studies.

Interestingly the shear bond strength of Biodentine increased significantly from 2 d to one week. This may be explained by the fact that that the setting reaction of CSCs might continue for more than a month [25]. After 7 d and 14 d there was no significant difference in the shear bond strength of Biodentine and the GIC ChemFill rock. Thus, it may be concluded from this result that Biodentine may be used for replacement of missing dentine in comparable indications to GIC. In contrast the shear bond strength of ProRoot MTA was significant lower at all investigation periods which may be interpreted as a clinical disadvantage of MTA concerning tooth restoration.

### Comparison to MTA

Identically to Biodentine to the best of our knowledge no data about shear bond strength of MTA to a planar prepared coronal dentine surface is available in literature. ProRoot MTA showed the lowest shear bond strength of all tested materials. A slight touch with the metal plunger was effectual to detach all ProRoot MTA samples from the dentine surface in the two days group. Nevertheless, the shear bond strength increased up to 5 MPa after two weeks. An increase of the shear bond strength values over time was also reported in push-out strength tests which range from 3.03 ± 1.28 MPa after 2 d [15] and between 4.75 ± 1.71 MPa [26] up to 9.0 ± 0.9 MPa [22] after 7 d, for example. Similar to the present study in general the push-out strength of Biodentine was higher compared to ProRoot MTA [22,23].

The different results of ProRoot MTA and Biodentine may be explained by the different particle size of these CSCs, which affects the penetration of cement into dentinal tubules in tag-like structures leading to a micromechanical anchor [17]. A smaller particle size and uniform components might have a role in better interlocking of Biodentine with dentine [15]. It was shown that calcium and silicon uptake into dentine leading the formation of tag-like structures in Biodentine was higher than in ProRoot MTA [17]. In contrast to ProRoot MTA the Biodentine-liquid contains calcium chloride as setting accelerator. It was discussed if the addition of calcium chloride may enhance the resistance of CSCs to displacement from dentine and thus to improve shear bond strength [20].

### Comparison to GIC

Since their introduction in 1972 [27], GICs have been widely used as dental restorative materials, luting cements and base materials [12,28]. One of their main advantages is the chemical bonding to tooth substrate by relative ease of use [29]. This is comparable to CSCs but there is a difference in the adhesive mechanism between acidic GIC and alkaline CSCs. The exact mechanism of GIC bonding to dentine is still unknown [12]. Several bonding mechanisms are discussed in literature: GIC bonds chemically directly to dentine by ionic bonding with hydroxyapatite to tooth substrate [30] even in presence of a smear layer [12]. Micromechanical bonding is also possible [12] and bonding to collagen has as well been suggested in a recent study [31]. The application of acidic GIC on dentine resulted in a demineralising effect on inorganic dentine components [16]. Polyalkenoic acids from the GIC is absorbed irreversibly onto hydroxyapatite from the dentine surface [32,33]. GIC forms an interaction zone by movement of ions from the cement into the surface layer of the tooth [21,34,35]. Hence, an ion exchange layer appears interfacial between dentine and GIC [36]. Whereas some authors reported about the formation of a hybrid layer and GIC tags in dentinal tubules after dentine conditioning and smear layer removal [37]. That could not be confirmed in other studies [38]. Hence, there was little evidence of tag-like structures when GIC is applied on dentine when the smear layer was not removed [16].

Bond strengths of GIC have been studied extensively using conventional shear testing methods but there is quite a bit of variation of the bond strengths of GIC on dentine surfaces that have not been conditioned [12,13,30,39]. For the GIC used in the present study (ChemFil rock) the manufacturer specified the shear bond strength to be 11.75 ± 4.75 MPa [40], which is in the range of the values found here.

### Comparison to X-trabase and Futurabond DC

The composite resin in combination with a dentine adhesive was included in this study as a positive control because it is well know that such materials possess the highest shear bond strength on dentine surfaces. Thus, the shear bond strength of X-trabase and Futurabond DC was about three times higher than that of GIC, Biodentine or ProRoot MTA. The present shear bond strength is in accordance with values found in literature for Futurabond DC ranging from 38.5 ± 14.8 MPa [41] to 39.30 ± 4.30 MPa [42] which confirms the accuracy of the method.

### Failure mode

The analysis of the failure modes showed that for Biodentine the predominant failure type was cohesive, while for ProRoot MTA most of the failures were adhesive between the filling material and the dentine interface. This finding is in agreement with previous studies evaluating ProRoot MTA [43-45] or comparing Biodentine and ProRoot MTA [14,15] in push-out tests.

As already discussed for the shear bond strength these results could be attributed to the different particle size of Biodentine and MTA, which affects the penetration of filling materials into dentinal tubules. Biodentine has a smaller particle size and uniform components that may contribute to better adhesion and interlocking with the dentine, which consequently results in cohesive failures within the filling material [15]. In addition, the ability of Biodentine to form tag-like structures into dentinal tubules increased the micromechanical attachment [15,16].

The microscopically observation revealed a predominantly cohesive failure mode within the tested GIC. These findings correspond to results previously reported [46-48] and it is believed that this cohesive failure occurs in GIC is due to the porosity within the cement itself. This porosity will act as a stress concentration point where the fracture will initiate [47]. It may be speculated in how far this will apply for Biodentine.

For the used composite resin and the dentine adhesive (X-trabase + Futurabond DC) the failure mode was determined to be 2/3 adhesive and 1/3 a mixture between adhesive and cohesive. This is not in fully accordance with the recent literature where a predominantly cohesive [41] or all failure modes (adhesive, cohesive, mixture) [42] were described for Futurabond DC.

## Conclusion

The null-hypotheses of this study could not be fully confirmed: Biodentine possess a shear bond strength to dentine comparable to a GIC, which was higher than that of ProRoot MTA but lower than that of composite resin in combination with a dentine adhesive. The shear bond strength of CSCs is increasing with time as the material cures.

Thus, under the aspect of dentine adhesion it may be concluded from the results of the present study that Biodentine may be used to replace missing dentine. Nevertheless, because the interaction of CSCs and the dentine surface is not fully understand yet further research concerning dentine-cement-interaction and dentine adhesion of CSCs is necessary.

## Abbreviations

CR: Composite resin
CSC: Calcium silicate cement
GIC: Glass ionomer cement
MTA: Mineral Trioxide Aggregate
